# Supplementary figures and images for: What’s the effectiveness of stocking actions in small creeks? The role of water discharge behind hatchery trout downstream movement
Source: PeerJ. 2022 Sep 26;10:e14069. doi: 10.7717/peerj.14069 (PMC9521347; doi:10.7717/peerj.14069)

# Residuals NFDD upstream

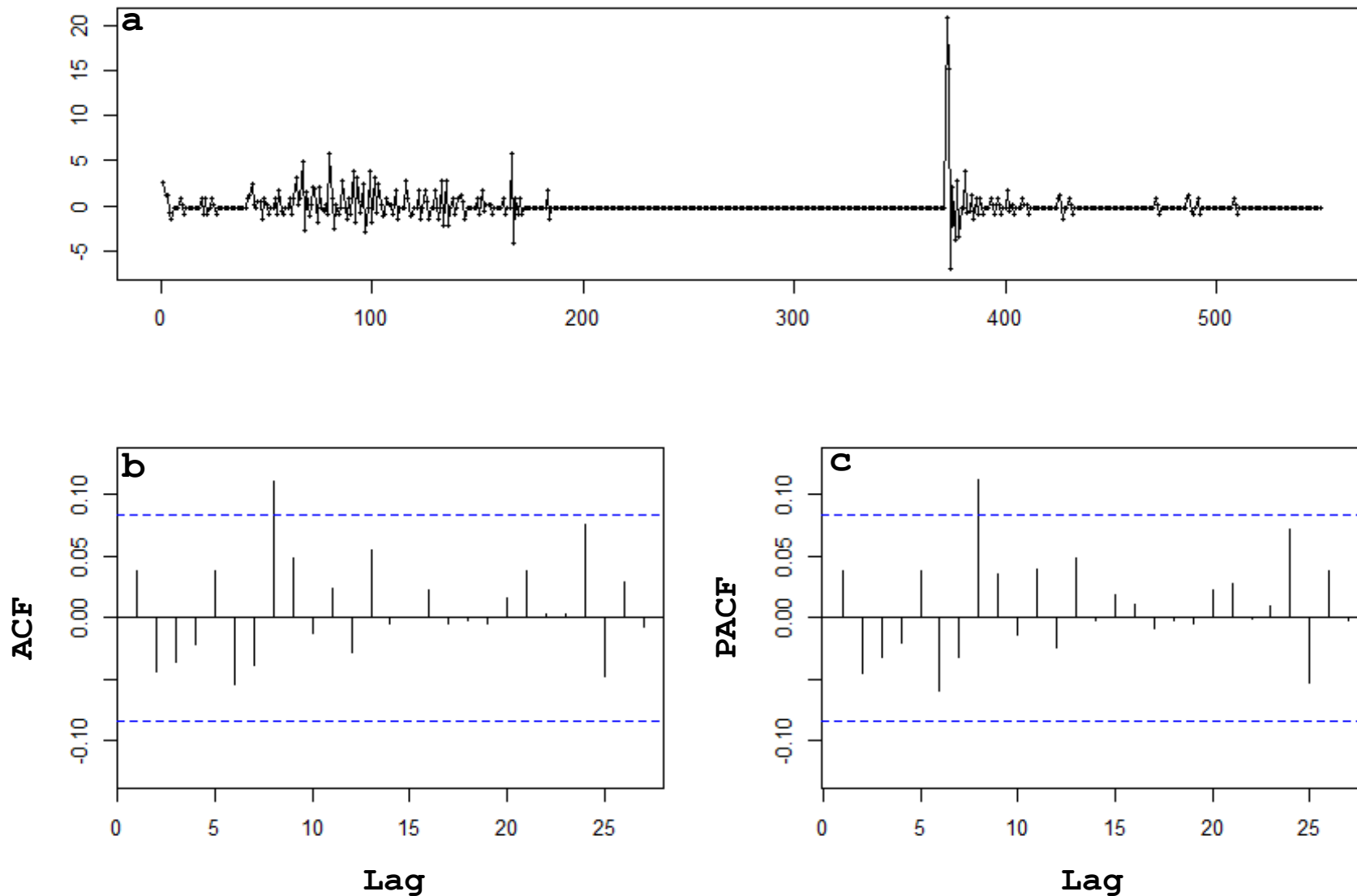

Supplement: Supplemental Information 1 — ARIMA model outputs for the time series of the number of fish detected daily (NFDD) released upstream the antenna displaying (a) plot of the model residuals, (b) plot of the autocorrelation function (ACF) and (c) plot of the partial autocorrelation function PACF. [file peerj-10-14069-s001.pdf]

# Residuals NFDD downstream

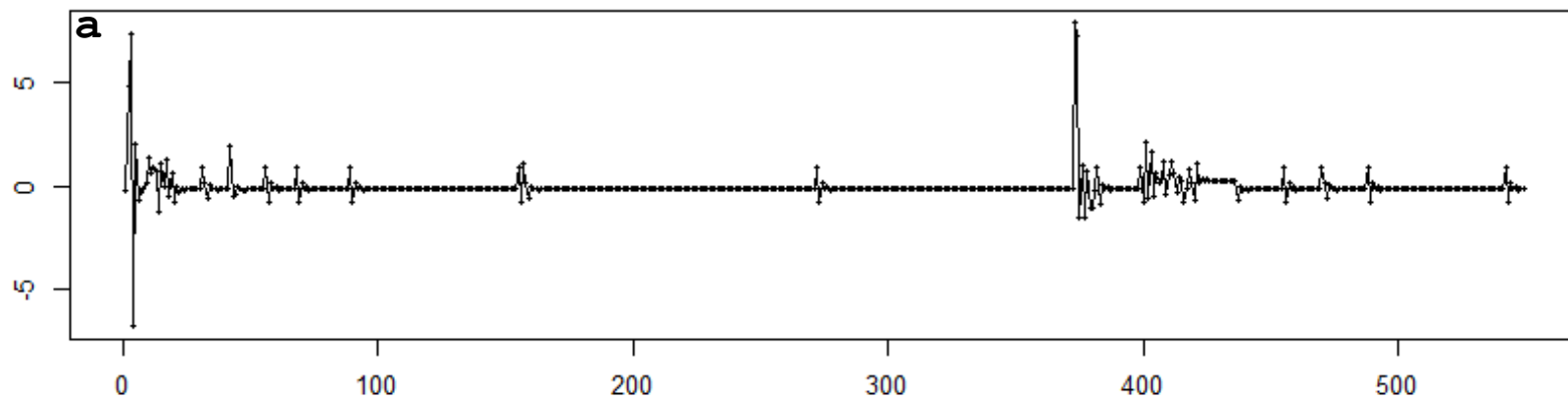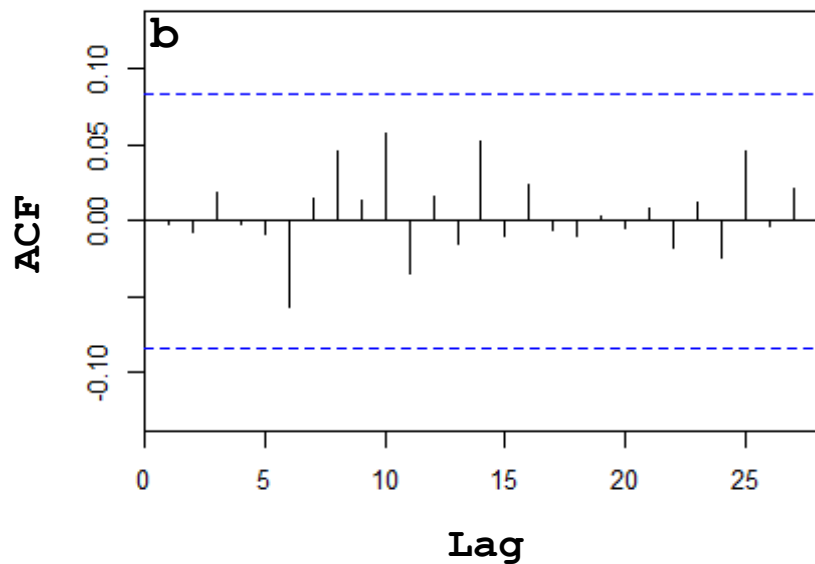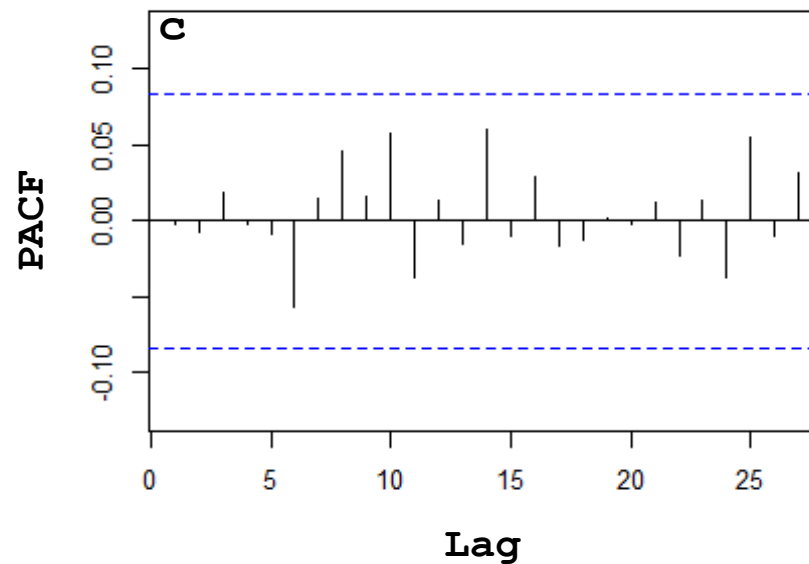

Supplement: Supplemental Information 2 — ARIMA model outputs for the time series of the number of fish detected daily (NFDD) released downstream the antenna displaying (a) plot of the model residuals, (b) plot of the autocorrelation function (ACF) and (c) plot of the partial autocorrelation function PACF. [file peerj-10-14069-s002.pdf]

# Residuals Water discharge

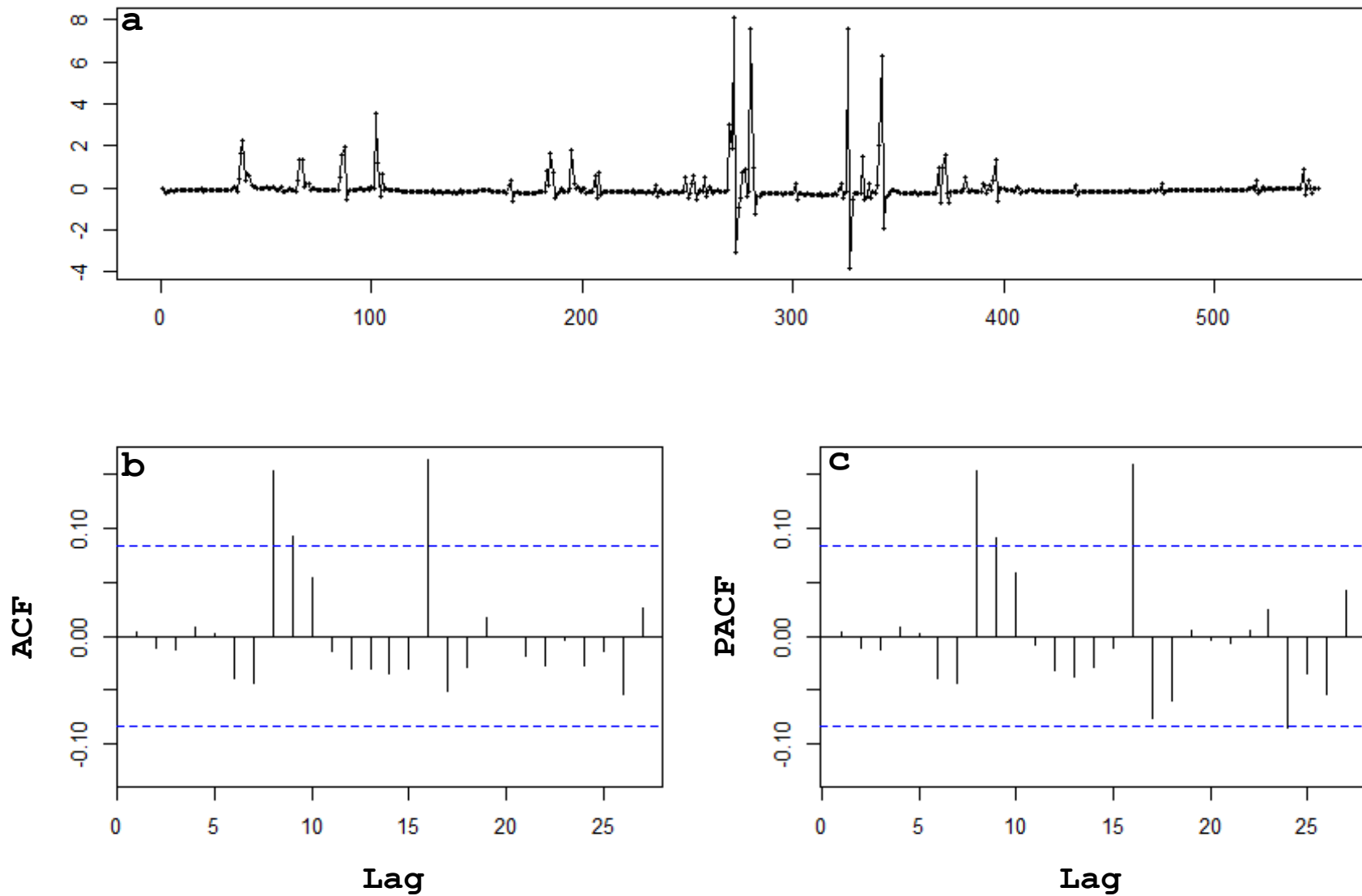

Supplement: Supplemental Information 3 — ARIMA model outputs of the daily water discharge time series displaying (a) plot of the model residuals, (b) plot of the autocorrelation function (ACF) and (c) plot of the partial autocorrelation function PACF. [file peerj-10-14069-s003.pdf]
